# Lack of efficacy of pomegranate supplementation for glucose management, insulin levels and sensitivity: evidence from a systematic review and meta-analysis

**DOI:** 10.1186/s12937-017-0290-1

**Published:** 2017-10-06

**Authors:** Haohai Huang, Dan Liao, Guangzhao Chen, Honglang Chen, Yongkun Zhu

**Affiliations:** 1 Department of Clinical Pharmacy, Dongguan Third People’s Hospital, Affiliated Dongguan Shilong People’s Hospital of Southern Medical University, Dongguan, Guangdong China; 2 Department of Gynaecology and Obstetric, Dongguan Third People’s Hospital, Affiliated Dongguan Shilong People’s Hospital of Southern Medical University, Dongguan, Guangdong China; 3 Department of Pharmacy, Guangdong Province Agricultural Reclamation Central Hospital, Zhanjiang, Guangdong China; 4 School of Pharmacy, Guangdong Medical University, Dongguan, Guangdong China

**Keywords:** Pomegranate, Glucose, Insulin, Diabetes mellitus, Meta-analysis

## Abstract

**Background:**

The potential glucose-lowering effects of pomegranate have been reported in animal and observational studies, but intervention studies in humans have generated mixed results. In this paper, we aimed to conduct a systematic review and meta-analysis of randomized controlled trials (RCTs) to evaluate the precise effects of pomegranate supplementation on measures of glucose control, insulin levels and insulin sensitivity in humans.

**Methods:**

Comprehensive electronic searches were conducted in PubMed, Embase, and the Cochrane Library. Studies included were RCTs that evaluated the changes in diabetes biomarkers among adults (≥18 years) following pomegranate interventions. The predefined outcomes included fasting blood glucose (FBG), fasting blood insulin (FBI), glycated haemoglobin (HbA1c), and homeostatic model assessment of insulin resistance (HOMA-IR). Endpoints were calculated as weighted mean differences (WMDs) with 95% confidence intervals (CIs) by using a random-effects model. Publication bias, subgroup analyses, sensitivity analysis and random-effects meta-regression were also performed to explore the influence of covariates on the net changes in fasting glucose and insulin concentrations.

**Results:**

Sixteen eligible trials with 538 subjects were included. The pooled estimates suggested that pomegranate did not significantly affect the measures of FBG (WMD, −0.6 mg/dL; 95% CI, −2.79 to 1.58; *P*=0.59), FBI (WMD, 0.29 μIU/mL; 95% CI, −1.16 to 1.75; *P*=0.70), HOMA-IR (WMD, −0.04; 95% CI, −0.53 to 0.46; *P*=0.88) or HbA1c (WMD, −0.11%; 95% CI, −0.39 to −0.18; *P*=0.46). Overall, significant heterogeneity was detected for FBI and HOMA-IR, but subgroup analysis could not identify factors significantly influencing these parameters. These results were robust in sensitivity analysis, and no significant publication bias was found in the current meta-analysis.

**Conclusion:**

Pomegranate intake did not show a notably favourable effect on improvements in glucose and insulin metabolism. The current evidence suggests that daily pomegranate supplementation is not recommended as a potential therapeutic strategy in glycemic management. Further large-scale RCTs with longer duration are required to confirm these results.

**Electronic supplementary material:**

The online version of this article (10.1186/s12937-017-0290-1) contains supplementary material, which is available to authorized users.

## Background

Diabetes mellitus is one of the most important public health challenges with an enormous economic burden worldwide. The estimated number of diabetic patients worldwide was 366 million in 2014, and it is projected to rise to 552 million by 2030 [1]. Glycemic control presents a constant challenge for patients with diabetes. Poor glycemic management causes long-term adverse outcomes in individuals with diabetes mellitus, including micro- and macrovascular complications such as myocardial infarction and stroke, renal failure, blindness and peripheral neuropathy [2–4]. Subjects with impaired fasting glucose (IFG) or impaired glucose tolerance (IGT) are usually considered to have a high future risk of developing diabetes [5]. Moreover, prospective cohort studies in subjects without diabetes have also revealed that increased insulin resistance worsened glycemic control and contributed to the development of type 2 diabetes mellitus (T2DM) [6–8]. Appropriate management of hyperglycaemia is thought to decrease the complications and morbidity of diabetes. Given its high disability and mortality rates, identifying modifiable lifestyle factors is important in primary, secondary, and tertiary prevention in patients with diabetes and healthy people. In recent decades, lifestyle interventions, including dietary micronutrients or functional food supplementation have generally been used to improve glycemic levels and have been incorporated into guidelines for the prevention and treatment of diabetes [9–11].

Pomegranate (*Punica granatum L.*) has a high concentration of antioxidants and bioactive polyphenols, and it has therefore been widely investigated for its antioxidant, anti-inflammatory, anti-atherogenic, and anti-hyperglycemic effects [12, 13]. Fresh pomegranate juice (PJ) is rich in phenolic acids (including gallic acid, caffeic acid, chlorogenic acid, ferulic acid, and coumaric acids), non-phenolic acids, citric acid, succinic acid, malic acid, oxalic acid, and ascorbic acid [14, 15]. Pomegranate seed oil (PSO) consists of approximately 80% conjugated linolenic acid, (9-cis, 11-trans, 13-cis) octadecatrienoic acid or punicic acid [16]. Pomegranate extract (PE) contains abundant anthocyanins, punicalin, pedunculagin, punicalagin, gallagic acid and ellagic acid [17]. Accumulating evidence indicates that pomegranate fractions from different parts of the fruit have been used to prevent and treat a wide range of diseases, including CVD, hypertension, obesity, and diabetes [18–20]. Encouraging findings from experimental research have indicated that pomegranate juice or pomegranate extracts improve glycemic metabolism, lower insulin requirements, and ameliorate insulin sensitivity [21–23]. Several observational studies have also suggested that pomegranate consumption was associated with improved glycemic control or a decreased risk for diabetes [24–26]. However, the precise effects of pomegranate on insulin and glucose metabolism in humans are inconsistent, and its optimal role in the clinical management of hyperglycaemia has not been fully established [27–32].

We performed a systematic review and meta-analysis of randomized controlled trials (RCTs) to assess the potential role of pomegranate treatment in the management of glycemic control, insulin levels and insulin sensitivity in comparison with placebo or other interventions among adults (≥18 years). Our primary outcomes were the differences in the levels of fasting blood glucose (FBG) and fasting blood insulin (FBI). Secondary outcomes included glycated haemoglobin (HbA1c) and homeostatic model assessment of insulin resistance (HOMA-IR).

## Methods

### Study eligibility criteria

Studies were eligible for inclusion in this review if they met the following criteria: 1) Study participants: adult male and female participants (age ≥ 18 y) with or without co-morbidities (such as hypertension, diabetes, or peripheral arterial disease) were included. 2) Types of interventions: participants needed to have specifically ingested a pomegranate intervention (no matter which type or regimen applied) for ≥1 week. Studies in which pomegranate was combined with other interventions (e.g., taking glucose-lowering drugs) were included when the control group received the same treatment. 3) Comparators: placebo or other interventions were used. 4) Outcome measures: studies reported data on at least one of the following endpoints: FBG, FBI, HbA1c, or HOMA-IR. In addition, the initial or endpoint values for the outcomes or their differences and their SD or SE or the 95% CI of each group were available. 5) Study design: each study was an RCT in humans with either a parallel or crossover design.

### Data sources and search strategy

We conducted and reported the present systematic review and meta-analysis following the PRISMA statement for the identification, screening, eligibility, and inclusion of articles [33]. Medline (http://www.ncbi.nlm.nih.gov/pubmed), Embase (http://www.embase.com), and the Cochrane Library (http://www.cochrane.org) databases were systematically searched for eligible studies from inception to February 28, 2017. Additionally, we also searched Google Scholar and ClinicalTrials.gov (https://clinicaltrials.gov/) to identify other potentially eligible trials. The structured search strategies used the following search key words and Medical Subject Headings (MeSH) terms: (pomegranate OR *Punica*) AND (glycemic control OR glycaemic control OR glucose control OR glycaemic OR glucose OR blood sugar OR blood glucose OR fasting plasma glucose OR FBG OR glucose tolerance OR insulin resistance OR insulin OR blood insulin OR fasting blood insulin OR insulin sensitivity OR FBI OR Haemoglobin A1c OR HbA1C OR glycated haemoglobin OR glycosylated haemoglobin OR homeostatic model assessment of insulin resistance OR HOMA-IR OR diabetes mellitus OR diabetes) AND sensitivity RCT filters (the specific and sensitive strategies developed to ensure optimal collection of RCTs in electronic searches). The search was restricted to English-language publications and clinical trials conducted in human subjects. Citation tracking was also performed on relevant review articles and editorials, and the reference lists of all the included studies were cross-referenced to ensure completeness. Two investigators (H.C. and D.L.) independently performed the literature searches. Any disagreements were resolved through discussion to reach consensus. After the removal of duplicate studies, we screened the titles and abstracts for relevance and accessed the full texts to identify the eligibility of the studies for inclusion in this meta-analysis.

### Data extraction

Data extraction was performed by D.L. and was confirmed independently by two other authors (H.H. and H. C.). Extracted data were entered into a predefined standardized Excel (Microsoft Corporation, USA) file. We also sought the supplementary files of the included trials or contacted the corresponding authors to verify the extracted data and to request any missing data. From each eligible trial, the following information was extracted: 1) Study characteristics, including first authors, publication year, sample size, study design, study duration, dose, type of intervention, and outcome measures. 2) Participants’ information, including mean age, sex, body mass index (BMI), baseline health status and baseline FBG. When the same patients were reported in several publications, we retained only the largest study to avoid duplication of information. For trials with more than one intervention group (e.g., with different doses of pomegranate), multiple comparisons were considered. The primary outcome measures were the net changes in FBG and FBI concentrations, and the secondary outcomes included changes in HbA1c and HOMA-IR. All values were captured as the means ± SD and converted to mg/dL for glucose and μIU/mL for insulin using the following conversion factors: 1 mmol/L = 18 mg/dL for glucose and 1 pmol/L = 6.945 μIU/mL for insulin value.

### Assessment of methodological quality

We used the Cochrane risk of bias tool to evaluate the risk of bias in the methodological quality of the included trials [34]. The assessment of quality characteristics used the following criteria: 1) sequence generation of allocation; 2) allocation concealment; 3) masking of participants and personnel; 4) blinding of outcome assessors; 5) incomplete outcome data; 6) selective outcome reporting; and 7) other sources of bias. Each item was judged to be at low, unclear, or high risk of bias, based on whether the level of bias in the domains may have led to material bias in the outcomes of interest. Trials with high risk of bias for any one or more key domains were considered to be at high risk of bias, while trials with low risk of bias for all key domains were considered to be at low risk of bias; otherwise, studies were considered to be at unclear risk of bias.

### Grading quality of evidence

Two reviewers (H. H. and D. L.) independently evaluated the quality of the evidence for the primary and secondary outcomes according to the GRADE methodology for risk of bias, inconsistency, indirectness, imprecision, and publication bias; they rated each as very low, low, moderate, or high. If disagreements occurred between the two reviewers, a third author would make the decision through discussion. Summary tables were constructed using the GRADE Profiler (GRADEpro, version 3.6).

### Statistical analysis

In the present meta-analysis, weighted mean difference (WMD) and 95% confidence interval (CI) were used as the main measures to summarize the clinical effect of the arms on the outcomes. We performed *I* ^*2*^ testing to assess the magnitude of the heterogeneity between trials; values greater than 50% were regarded as being indicative of moderate-to-high heterogeneity [35]. We pooled outcome data using a random-effects model accounting for clinical heterogeneity [36]. To explore the possible influence of covariates on net changes, pre-specified subgroup analyses were conducted to evaluate the effects of the following factors on primary outcomes: pomegranate-product types, study design, baseline FBG, study duration, health status and initial BMI measurements of the participants. In addition, we further explored our findings using two additional sensitivity analyses. To assess the potential impact of the quality of the studies on the outcomes, we performed a sensitivity analysis with the exclusion of low-quality studies. To test the robustness of the findings, we also conducted a sensitivity analysis, which was investigated using the leave-one-out approach (omitting one study at each turn and repeating the analysis). We plotted the SEs of the studies against their corresponding effect sizes to examine potential publication biases in the meta-analysis. Publication bias was assessed by visually inspecting a funnel plot and by using the Egger test [37]. A *P* value of <0.05 was considered statistically significant for all analyses. All statistical analyses were performed using STATA (version 12; StataCorp, College Station, TX).

### Meta-regression analysis

As potential confounders of treatment effects, dosage and duration of supplementation with pomegranate juice were applied by a restricted maximum likelihood (REML) based meta-regression analysis to explore their association with the estimated effect size in glucose outcome [38]. The analysis was also performed by using STATA version 12.0 (Stata Corporation LP, College Station, TX, USA). This method corresponds to random-effects meta-regression including both within-study variances of treatment effects and the residual between-study heterogeneity.

## Results

### Identification of relevant studies

**Fig. 1:**
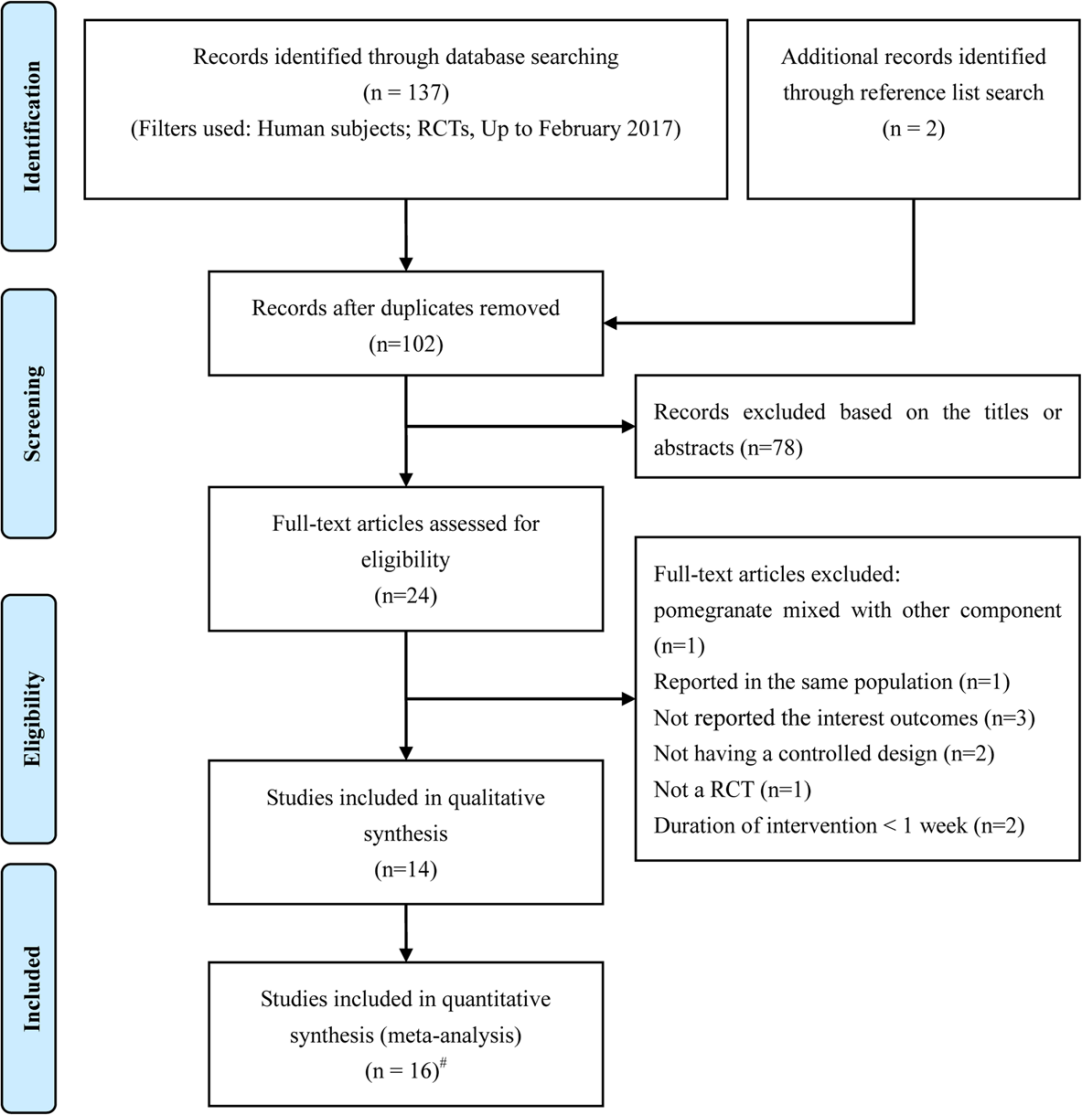
Flow chart of data base searches and articles included in the present meta-analysis. ^#^ The work conducted by Heber et al. was separated into 2 trials; The study conducted by Fuster-Munoz et al. was also separated into 2 trials

Fig. 1 shows the details of our literature screening, study selection, and reasons for exclusion. The initial search yielded 139 potentially relevant citations. After the removal of duplicates, 102 titles and abstracts were screened; of these, 78 were excluded because they were clearly not relevant to our meta-analysis. The full-text publications were obtained for the remaining 24 articles. A total of 10 articles were subsequently excluded for the reasons listed in Fig. 1. Subjects in one study were also divided into 2 subgroups on the basis of different doses of pomegranate ellagitannin extract consumption used (710 mg/day intake subgroup and 1420 mg/day intake subgroup) [39]. The work conducted by Fuster-Munoz et al. was also separated into 2 subgroups: the pomegranate juice and the pomegranate juice diluted 1:1 with water subgroups [40]. Finally, a total of 16 RCTs that met our inclusion criteria were included in the present pooled analysis [27–32, 39–46].

### Study characteristics

**Fig. 2:**
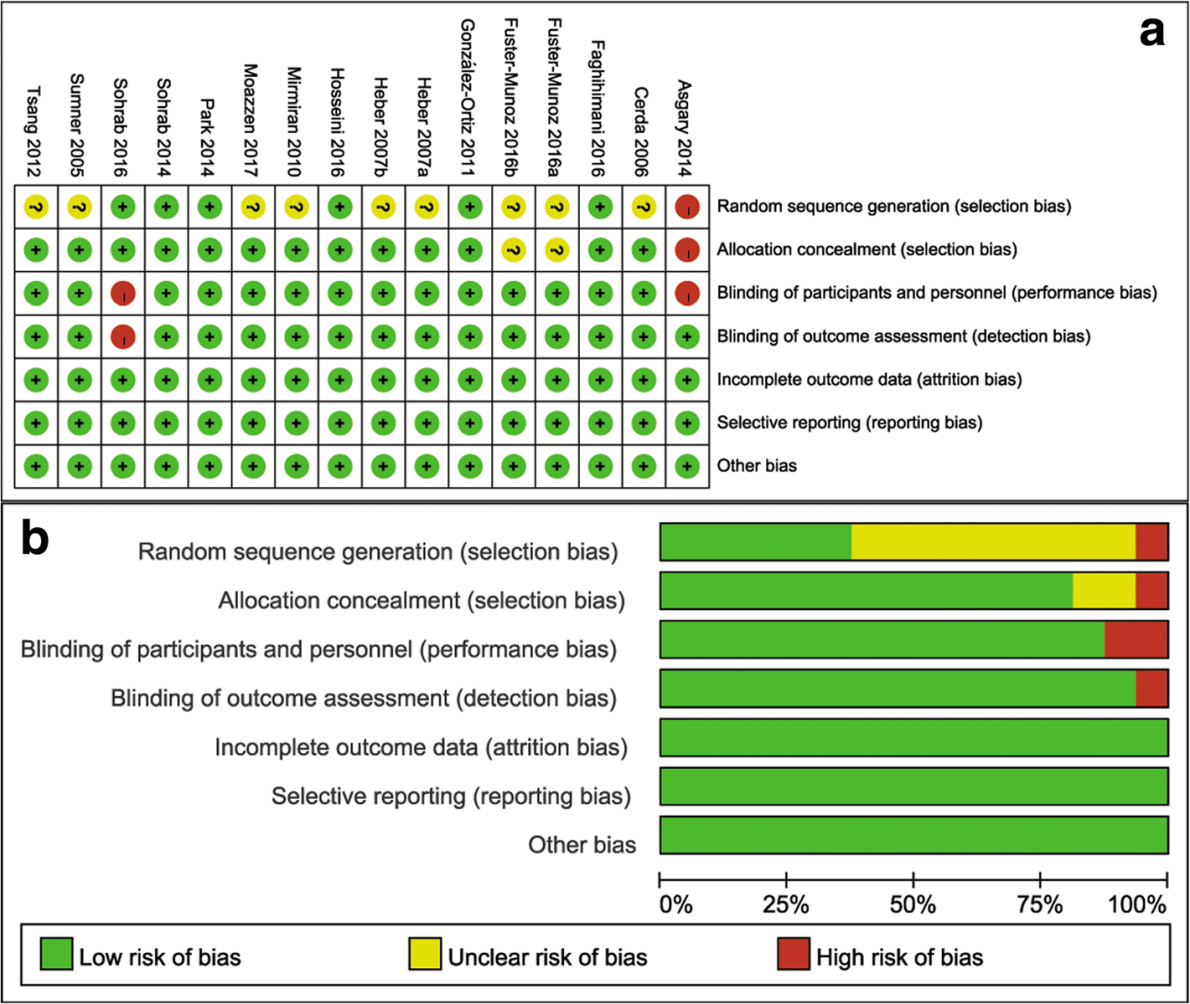
Results of risk of bias assessment. **a** Risk of bias graph: review authors’ judgments about each risk of bias item presented as percentages across all included studies. **b** Risk of bias summary: review authors’ judgments about each risk of bias item for each included study

A summary of the study characteristics included in the meta-analysis are presented in Table 1. Sixteen trials with a total of 627 subjects were included in the meta-analysis. The total number of subjects included in each study ranged from 14 to 74 subjects. Among the included studies, fifteen studies reported the FBG outcome [27, 28, 30–32, 39–46], 8 reported FBI values [29–31, 41–43, 45, 46], 3 reported HbA1c [27, 41, 43] and 7 reported HOMA-IR [29, 31, 41–43, 45, 46]. The mean age of participants in each trial ranged from 30 to 70 years, with differing age ranges in most studies. Of the 16 trials included in the current meta-analysis, 11 studies used pomegranate juice as a supplement (the dosage ranged from 120 to 500 ml/day) [27, 28, 30–32, 40, 41, 44–46], 2 studies used pomegranate seed oil as treatments (the dosage ranged from 400 to 2000 mg/day) [29, 43], and 3 studies utilized pomegranate extract as the intervention (the dosage ranged from 710 to 1420 mg/day) [39, 42]. The duration of the pomegranate intervention varied from 1 to 12 week (median: 5.5 week). Twelve of the 16 studies included subjects with a risk of cardiovascular disease, such as subjects with T2DM, overweight or obesity, hypertension, hyperlipidaemia and metabolic syndrome. Of the remaining 4 trials, one was conducted in patients with stable chronic obstructive pulmonary disease, and 3 studies were performed in healthy subjects. Fourteen trials adopted parallel study designs, and the 2 remaining trials used crossover designs. Fourteen trials were double-blind, placebo-controlled clinical trials. Among the included studies, investigators attempted to maintain the usual lifestyles of the participants. Details regarding the risk of bias assessment for the individual validity components are presented in Fig. 2. Overall, 5 trials were categorized as at low risk of bias, 2 as a high risk of bias, and 9 as unclear.

**Table 1:** Characteristics of 16 randomized controlled trials included in analysis^a^

Author, publication year	Sample Size	Study design	Details of participants selection	Sex (M/F)	Age (years)^b^	BMI (kg/m^2^)^b^	Intervention group	Control group	Duration (weeks)	Location	FBG at baseline (T vs. C)^b^	Outcomes of measures
Sumner, 2005	45	R, DB, P	Patients with coronary heart disease	40/5	69.0 ± 10.0	28.5 ± 5.6	PJ consumption; 240 ml/day	Placebo (modified sports beverage)	12	USA	T: 113.0 ± 30.0 C: 116.0 ± 51.0	FBG, HbA1_C_
Cerda, 2006	30	R, DB, P	Patients with stable chronic obstructive pulmonary disease	NA	61.7 ± 10.0	31.0 ± 5.4	PJ consumption; 400 ml/day	Placebo	5	Spain	T: 114.5 ± 25.5 C: 113.7 ± 51.5	FBG,
Heber, 2007a	42	R, DB, P	Overweight individuals with increased waist size	NA	40–70	33.5 ± 8.5	PE extract; 710 mg/day	Placebo	4	USA	T1: 95.3 ± 10.4 T2: 89.2 ± 7.6	FBG,
Heber, 2007b	41	R, DB, P	Overweight individuals with increased waist size	NA	40–70	33.5 ± 8.5	PE extract; 1420 mg/day	Placebo	4	USA	T: 89.2 ± 7.6 C: 97.8 ± 9.0	FBG,
Mirmiran, 2010	45	R, DB, P	Subjects with hyperlipidaemic	NA	53.0 ± 9.0	27.7 ± 3.4	PSO consumption; 400 mg/day	Placebo	4	Iran	NA	FBI, HOMA-IR
González-Ortiz, 2011	20	R, DB, P	Patients with obesity	NA	37.3 ± 9.5	34.5 ± 3.7	PJ consumption; 120 ml/day	Placebo	4	Mexico	T: 84.6 ± 5.4 C: 84.6 ± 9.0	FBG, FBI
Tsang, 2012	28	R, DB, C	Healthy volunteers	12/16	50.4 ± 6.1	26.8 ± 3.4	PJ consumption; 500 ml/day	Placebo (water plus the equivalent carbohydrates)	4	UK	T: 88.0 ± 6.7 C: 83.7 ± 8.6	FBG, FBI, HOMA-IR
Asgary, 2014	21	R, SB, P	Subjects with hypertension	6/15	52.9 ± 10.8	27.4 ± 3.8	PJ consumption; 150 ml/day	Placebo (water)	2	Iran	T: 90.1 ± 6.1 C: 87.8 ± 10.9	FBG, FBI
Park, 2014	77	R, DB, P	Overweight women	0/77	41.5 ± 12.5	28.4 ± 2.2	PJ consumption; 200 ml/day	Placebo (beverage)	8	Korea	T: 98.5 ± 8.2 C: 97.1 ± 9.4	FBG, FBI, HOMA-IR
Sohrab, 2014	44	R, DB, P	Patients with T2DM	NA	55.9 ± 6.7	29.0 ± 4.0	PJ consumption; 250 ml/day	Placebo	12	Iran	T: 160.3 ± 47.8 C: 148.7 ± 42.1	FBG, FBI, HOMA-IR, HbA1_C_
Hosseini, 2016	42	R, DB, P	Overweight and obese individuals	NA	30–60	31.8 ± 4.5	PE supplementation; 1000 mg/day	Placebo	4	Australia	T: 98.6 ± 10.4 C: 99.6 ± 14.9	FBG, FBI, HOMA-IR
Faghihimani, 2016	74	R, DB, P	Patients with T2DM	26/52	50.0 ± 6.8	26.5 ± 2.6	PSO capsules consumption; 2000 mg/day	Placebo(medium chain triacylglycerol)	8	Iran	T: 149.0 ± 39.0 C: 156.0 ± 56.0	FBG, FBI, HOMA-IR, HbA1_C_
Sohrab, 2016	60	R, SB, P	Patients with T2DM	NA	54.7 ± 8.4	27.3 ± 3.7	PJ consumption; 200 ml/day	Placebo	6	Iran	T: 158.1 ± 41.1 C: 193.0 ± 63.3	FBG,
Fuster-Munoz, 2016a	14	R, DB, P	Endurance-based athletes	14/0	35.3 ± 8.8	NA	PJ consumption; 200 ml/day	seasonal fruit	3	Spain	T: 79.0 ± 8.0 C: 74.4 ± 9.5	FBG,
Fuster-Munoz, 2016b	14	R, DB, P	Endurance-based athletes	14/0	35.3 ± 10.4	NA	PJ diluted 1:1 with water consumption; 200 ml/day	seasonal fruit	3	Spain	T: 73.3 ± 8.0 C: 74.4 ± 9.5	FBG,
Moazzen, 2017	30	R, DB, C	Patients with metabolic syndrome	13/17	51.57 ± 10.0	NA	PJ consumption; 500 ml/day	Placebo	1	Iran	T: 144.6 ± 11.9 C: 144.7 ± 12.3	FBG, FBI, HOMA-IR

^a^ *R* randomized, *DB* double-blind, *SB* single-blind, *C* crossover, *P* paralle, *F* female, *M* male, *NA* not available, *BMI* body mass index, *PE* pomegranate ellagitannin, *PJ* pomegranate juice, *PSO* pomegranate seed oil, *T* Treatment group, *T2DM* type 2 diabetes mellitus, *C* control group, *FBG* fasting blood glucose, *FBI* fasting blood insulin, *HbA1c* glycated hemoglobin, *HOMA-IR* homeostatic model assessment of insulin resistance

^b^Values are provided as mean ± SD, except the studies conducted by Heber et al. and Hosseini et al. The values in these studies were

### Overall effect of pomegranate on glucose control and insulin sensitivity

**Fig. 3:**
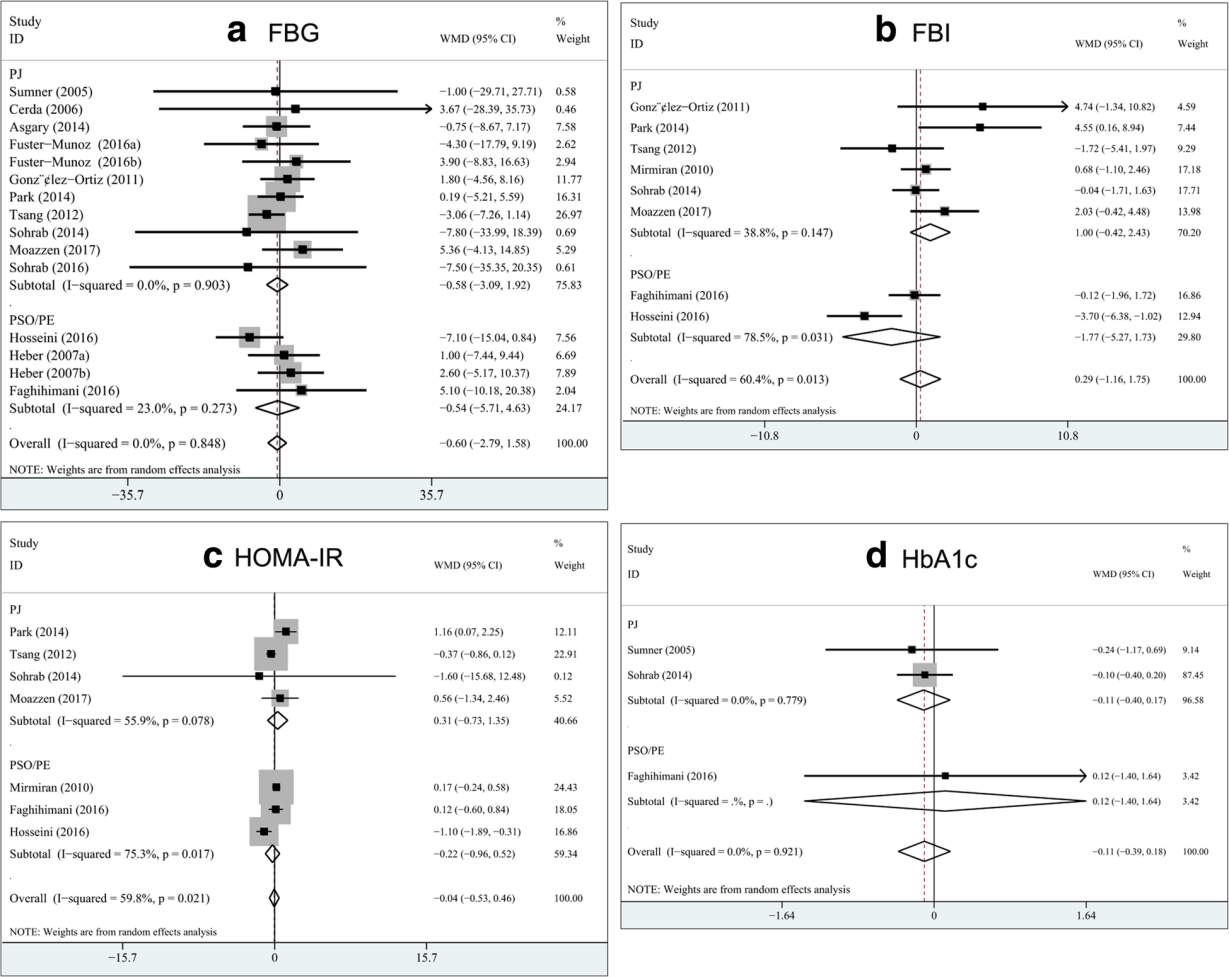
Pooled estimated effect of pomegranate on glucose control and insulin sensitivity as compared with the control arms. **a** fasting blood glucose; **b** fasting blood insulin, **c** homeostatic model assessment of insulin resistance; **d** glycated hemoglobin. WMD, weighted mean difference; PJ, pomegranate juice; PSO, pomegranate seed oil; PE, pomegranate extract

A total of 15 trials reported data on FBG concentrations. Compared with the control group, pomegranate intake did not significantly affect the FBG concentrations, and the pooled estimated change in FBG was −0.60 mg/dL (95% CI: -2.79, 1.58; *P* = 0.59) by the random-effects model (Fig. 3a). Eight trials reported data on fasting insulin concentrations, and the pooled estimated effect was 0.29 μIU/mL (95% CI: -1.16, 1.75; *P* = 0.70) by the random-effects model (Fig. 3b). Seven studies reported the results on HOMA-IR, and the pooled results from the random-effects model for estimated mean difference was −0.04 (95% CI: -0.53, 0.46; *P* = 0.88) (Fig. 3c). Three studies provided data on HbA1c, and pooling the data of these studies showed that pomegranate supplementation failed to show a significant effect on HbA1c (random-effects model; WMD, −0.11; 95% CI, −0.39, 0.18; *P* = 0.46; Fig. 3d). Between-study heterogeneity was observed in the effects of pomegranate on fasting insulin concentrations (I^2^ = 60.4%) and HOMA-IR (I^2^ = 59.8%), but not in HbA1c (I^2^ = 0%) or FBG (I^2^ = 0%) levels.

### Subgroup analyses and sensitivity analysis

Subgroup analyses were performed to explore the effects of health status (CVD risk vs. healthy), study design (parallel vs. crossover), types of intervention (PJ supplementation vs. PE/POS supplementation), intervention duration (<5 week. vs. ≥5 week), baseline FBG levels (<100 mg/dL vs. 100–126 mg/dL vs. ≥126 mg/dL) and baseline BMI (<30 kg/m^2^ vs. ≥30 kg/m^2^) on the overall effects of pomegranate on fasting glucose and insulin concentrations, respectively.

The subgroup analysis results are summarized in Table 2 **.** There were no statistically significant differences in the pooled effects of pomegranate on FBG in the subgroups stratified by study designs, intervention durations, types of intervention, baseline BMI, and baseline FBG levels. We also stratified the studies according to the type of patient, and no significant difference in the FBG-lowering effect was found between trials that were conducted in subjects with CVD risk (WMD, 0.30 mg/dL, 95% CI, −2.36 to 2.97; *P* = 0.82) and those that were conducted in healthy individuals (WMD, −2.53 mg/dL, 95% CI, −6.36 to 1.30; *P* = 0.19). Similarly, the subgroup analyses indicated that differences in study design, type of intervention, baseline BMI, and health status of the participants did not appear to significantly influence pooled mean differences in FBI concentrations.

**Table 2:** Subgroup analyses of fasting glucose and insulin concentrations stratified by previously defined study characteristics

Variables	Fasting glucose				Fasting insulin			
	No. of trials	Net change (95% CI)^a^	*P* ^*^	I^2^ (%)^b^	No. of trials	Net change (95% CI)^a^	*P* ^*^	I^2^ (%)^b^
Sensitivity analyses								
Exclude high-risk research	13	−0.55 (−2.82 to 1.73)	0.64	0	8	0.29 (−1.16 to 1.75)	0.70	60
Removing study did not use placebo as control	13	−0.64 (−2.89 to 1.60)	0.58	0	8	0.29 (−1.16 to 1.75)	0.70	60
Subgroup analyses								
Study design								
Parallel	13	−0.09 (−2.74 to 2.56)	0.95	0	6	0.24 (−1.49to 1.97)	0.78	65
Crossover	2	0.03 (−7.92 to 7.98)	0.99	60	2	0.42 (−3.22 to 4.06)	0.82	64
Duration								
<5 week	9	−0.82 (−3.27 to 1.63)	0.51	0	5	0.01 (−2.40 to 2.42)	0.99	71
≥ 5 week	6	0.22 (−4.57 to 5.02)	0.93	0	3	0.58 (−1.28 to 2.43)	0.54	49
Type of intervention								
PJ consumption	11	−0.58 (−3.09 to 1.92)	0.65	0	5	1.28 (−0.74 to 3.30)	0.22	51
<250 ml/d	7	0.32 (−3.03 to 3.67)	0.85	0	3	2.86 (0.00 to 4.88)	0.05	0
≥ 250 ml/d	4	−1.73 (−5.51 to 2.04)	0.37	0	2	−0.33 (−1.85 to 1.20)	0.67	0
PE/PSO consumption	4	−0.67 (−5.10 to 3.77)	0.77	23	3	−0.84 (−3.12 to 1.44)	0.47	73
≤ 1000 mg/d	2	−3.30 (−9.08 to 2.48)	0.26	47	2	−1.39 (−5.68 to 2.90)	0.52	86
>1000 mg/d	2	3.31 (−3.81 to 10.04)	0.38	0	1	−0.12 (−1.96 to 1.72)	0.9	NA
Healthy status								
CVD risk	11	0.30 (−2.36 to 2.97)	0.82	0	7	0.51 (−1.05 to 2.07)	0.52	64
Healthy	3	−2.53 (−6.36 to 1.30)	0.19	0	1	−1.72 (−5.41 to 1.97)	0.36	NA
BMI								
<30 kg/m^2^	7	−1.56 (−4.51 to 1.39)	0.30	0	5	0.29 (−0.87 to 1.44)	0.63	24
≥ 30 kg/m^2^	5	−0.10 (−3.83 to 3.62)	0.96	0	2	0.06 (−8.16 to 8.28)	0.99	84
Baseline FBG levels								
<100 mg/dL	9	−1.00 (−3.29 to 1.30)	0.39	0	4	0.51 (−3.74 to 4.76)	0.81	78
100–126 mg/dL	2	1.08 (−20.31 to 22.47)	0.92	0	0	–	–	–
≥ 126 mg/dL	4	3.33 (−4.1 to 10.75)	0.38	0	3	0.38 (−0.81 to 1.56)	0.53	12

^a^Net change was expressed as weighted mean difference

^b^The I^2^ statistic was calculated by using Cochran’s test, and I^2^>50% was considered to indicate significant heterogeneity across studies

^*^ *P* for meta-analysis: *P*<0.05 was considered to indicate a significant effect of pomegranate on fasting glucose and insulin concentrations by using a random-effects model

To further confirm the robustness of the results, we conducted a sensitivity analysis. Exclusion of any individual study from the overall analysis did not meaningfully change the magnitude or direction of the summary effect of pomegranate on fasting glucose and insulin concentrations. Sensitivity analyses also showed that the aggregated results in FBG was not altered after removal of the 2 trials with a high risk of bias, and 1 trial did not use a placebo as a control (Table 2).

### Meta-regression analysis of the dose-response effect

**Fig. 4:**
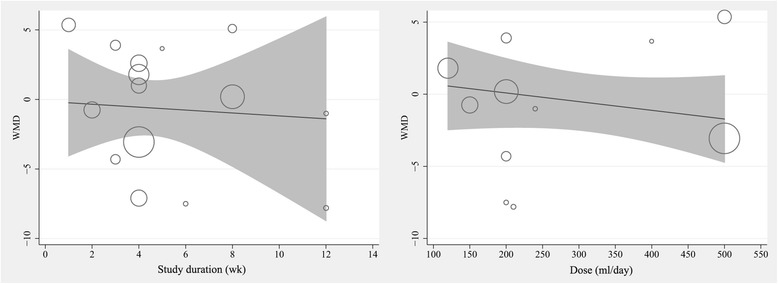
Meta-regression result of the association between mean changes in fasting glucose concentrations with dose and duration of supplementation with pomegranate juice

The meta-regression  analyses were performed only for FBG because of the small numbers of studies including the other outcomes. Here, random-effects meta-regression analysis suggested that no significant association was observed between the duration of pomegranate supplementation and mean differences in glucose levels (coefficient, −0.119; 95% CI, −1.29 to 1.06; *P* = 0.83). Moreover, our meta-regression analysis showed that the factor (dose of pomegranate juice supplementation) was not associated with the treatment effects on FBG level (coefficient, −0.006; 95% CI, −0.023 to 0.011, *P* = 0.46). The results of the meta-regression analyses are shown in Fig. 4.

### Publication bias and GRADE profile evidence

**Fig. 5:**
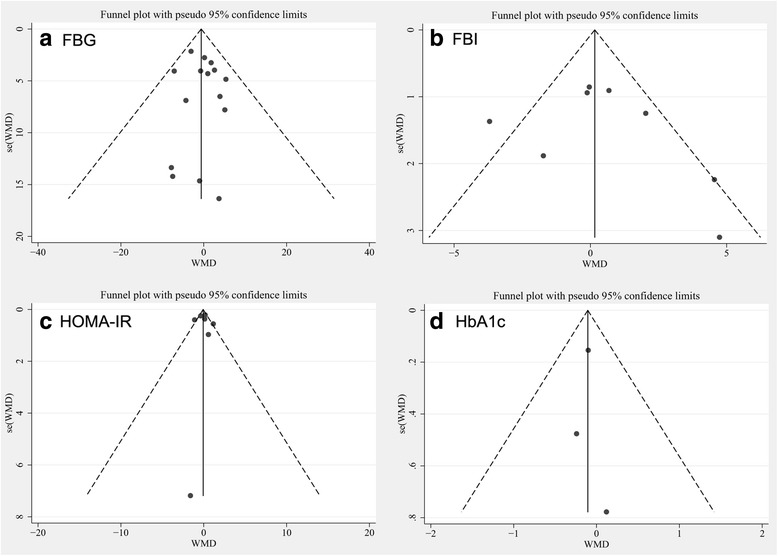
Tests for publication bias of impact of pomegranate consumption on glucose control and insulin sensitivity. **a** FBG, fasting blood glucose; **b** FBI, fasting blood insulin; **c** HOMA-IR, homeostatic model assessment of insulin resistance; **d** HbA1C, glycated hemoglobin

No evidence of publication bias was found in the present meta-analysis (for both primary and secondary outcomes). The funnel plots were symmetrical (Fig. 5), and Egger’s regression test suggested no significant asymmetry for the overall effect estimation on the study endpoints (for FBG, *P* = 0.96; for FBI, *P* = 0.62; for HOMA-IR, *P* = 0.88; for HbA1c, *P* = 0.60). The GRADE evidence profiles for the primary and secondary outcomes are presented as Additional file 1. The GRADE Working Group grades of the level of evidence are high for FBG and HbA1c and moderate for FBI and HOMA-IR.

## Discussion

The findings from the current study demonstrate that pomegranate intake did not lead to any significant changes in circulating glucose levels, insulin concentrations, HOMA-IR or HbA1c. The target population for T2DM prevention has been a topic of debate since the completion of major diabetes prevention trials [47]. The difficulty stems from observations that diabetes prevalence has increased across all segments of society [48]. However, the evidence for preventive interventions is mainly limited to persons with impaired glucose tolerance (IGT) [49]. Assessment of glycemic control is a key element of diabetes care. Reliable information about glycemic variations allows physicians and patients to evaluate the effect of treatment on restoration and maintenance of blood glucose to within the physiologic range.

Dietary micronutrients or herbal medication interventions have generally been used to improve glycemic control and other CVD risk factors among individuals with T2DM, and the public has embraced their efficacy and safety [11, 50, 51]. A recent meta-analysis reported that consumption of cinnamon is associated with a statistically significant decrease in the levels of FBG, total cholesterol, LDL-C, and triglyceride levels and an increase in HDL-C levels [11]. Potential side effects reported to be associated with cinnamon were hepatotoxicity, decreased platelet counts, increases in the risk of bleeding, and markedly increased allergy/hypersensitivity. However, human studies suggested that no significant side effects were seen with cinnamon use. Hausenblas et al. [50] found that resveratrol supplementation was more effective than placebo in controlling systolic blood pressure, HbA1c, and creatinine. The incidence of side effects was very small and not different from placebo, and no major adverse events were reported.

Over the last decade, various studies have linked pomegranate and its active compounds with diabetes prevention and treatment. The study conducted by Huang et al. demonstrated that treatment with *Punica granatum* flower extract could enhanced the mRNA expression of cardiac PPAR-γ and restore the mRNA expression of the cardiac glucose transporter 4 (GLUT4) [52]. Later reports have shown that PSO reduced the risk for T2DM in wild-type CD-1 mice by improving insulin sensitivity [53]. Pomegranate flowers also ameliorate T2DM in Zucker fatty diabetic rats by enhancing the expression of hepatic genes involved in fatty acid oxidation (e.g., acyl-CoA oxidase and carnitine palmitoyl-transferase-1) [54]. Furthermore, pomegranate extract is beneficial in controlling glucose homeostasis in humans by suppressing the activation of NF-κB, neutralizing the generated reactive oxygen species and the expression of tumour necrosis factor-α, which finally delays the development of T2DM [55, 56].

However, data from human clinical trials that have evaluated the possible beneficial effects of pomegranate products and extracts on glycemic control and insulin sensitivity have generated mixed findings [27–32]. In the present study, we aim to provide a focused and comprehensive evaluation of the precise effects of pomegranate supplementation on measures of glycaemic control and insulin sensitivity in humans. A total of 16 RCTs were included in the analysis, and methodological quality assessment suggested that the overall data quality was fair. The Egger’s regression test’s symmetry testing of the funnel plot did not indicate a notable publication bias for the overall effect estimation of WMDs in the primary outcomes. The results remain robust and consistent when pre-specified defined subgroup analyses were conducted. Meta-regression analysis also revealed the absence of any dose-response relation between the pomegranate juice intake and any effect on FBG concentration. Moreover, exclusion of any single study and sensitivity analyses based on various exclusion criteria (i.e., removal of the trials with a high risk of bias and the trials that did not use a placebo as a control) did not materially alter the pooled results, which adds robustness to our main results. The magnitude of WMDs reported in high-quality studies was stronger than the magnitude reported in the overall analysis (0.55 mg/dL compared with 0.06 mg/dL), which indicated that the real effects may be influenced by poor study methodologies.

Several methods, each with differing utility and limitations, exist for monitoring glycemic control [57]. Fasting plasma glucose levels are considered a key variable in the diagnosis of diabetes and are also adopted by the FDA to evaluate the efficacy of dietary supplements and drugs. Among the included studies, most studies reported only fasting glucose (93.7%) and fasting insulin (50%) concentrations, and there was a lack of other important variables for glycemic control and insulin sensitivity. Previous data indicated that glucose and insulin concentrations fluctuate with changes after diet, exercise, and use of some medications. Glycated protein, such as HbA1c, is considered the standard measure of long-term glycemic control, and the measurement of HbA_1c_ levels is strongly associated with complications of diabetes. In the present study, however, we failed to find any statistically significant differences between the relation between dietary pomegranate supplementation and reductions in HbA1c (WMD, −0.11%, 95% CI, −0.39 to 0.18, *P* = 0.46). Most notably, these results are inconclusive because of the limited eligible RCTs included in these outcomes (only 3 studies reported HbA1c concentrations); further large-scale and well-performed studies that report a more comprehensive set of indicators for glycemic control and insulin sensitivity are needed.

Our meta-analysis has several strengths. The present analysis was restricted to RCTs that met predetermined methodological criteria to minimize the potential for bias. The relatively large number of pooled participants allowed us a greater statistical power to detect a small treatment effect. To increase the robustness of our study, we performed several subgroup analyses and sensitivity analysis. Although we had limited this study to well-designed RCTs and performed quality assessment to reduce the possible selective bias, the present meta-analysis still had several caveats that could affect the interpretation of the results. First, although extensive searches with clear inclusion criteria were performed, it cannot be entirely guaranteed that all relevant articles were included, since the measures of blood glucose or insulin were not primary outcomes in the trials selected for this meta-analysis, and the null findings of secondary outcomes may not always have been published. Second, there was unavoidable heterogeneity across the studies for FBI and HOMA-IR outcomes (I^2^ = 60.4% and 59.8%, respectively). This heterogeneity may have been due to study differences in design, study population and characteristics, and duration of treatment. Regardless of the cause, the pooled multivariate estimates were managed using a random-effects model, which could reduce the bias to some extent. Third, our pooled results were based on unadjusted estimates; the precise effect of pomegranate on diabetes biomarkers could be impacted by various confounders (i.e., other lifestyle interventions or smoking habits). The synergistic effects of other coexisting substances on the clinical outcomes need to be excluded during the study period. Fourth, due to the limited number of DM patients included, the overall effects of pomegranate on parameters of glucose metabolism are inconclusive; additional adequately powered studies investigating the effects of pomegranate on the biomarkers of glucose metabolism in adults with DM are needed. Finally, the intervention durations of the included studies were relatively short (ranging from 1 to 12 weeks with a median of 5.5 weeks), and the more long-term durability of the pomegranate treatment therefore is unknown. Finally, a standardized protocol of pomegranate consumption (such as consistency regarding dosage, route, timing, and duration of the intervention) are needed in the future.

## Conclusions

In conclusion, pomegranate intake did not show notably favourable effects on improvements in glucose and insulin metabolism. The current evidence suggests that daily pomegranate supplementation is not recommended as a potential therapeutic strategy in glycemic and insulin management. Additional well-reported RCTs that are specifically designed to evaluate the effect of pomegranate or pomegranate extracts on a set of comprehensive clinical outcomes related to glycemic control are required.

## Additional file

Additional file 1: GRADE Evidence Profile. (DOC 38 kb)

## Abbreviations

CI: Confidence interval
CVD: Cardiovascular disease
FBG: Fasting blood glucose
FBI: Fasting blood insulin
HbA1c: Glycated hemoglobin
HOMA-IR: Homeostatic model assessment of insulin resistance
IFG: Impaired fasting glucose
IGT: Impaired glucose tolerance
MeSH: Medical Subject Headings
PE: Pomegranate extract
PJ: Pomegranate juice
PRISMA: Preferred Reporting Items for Systematic Reviews and Meta-Analyses
PSO: Pomegranate seed oil
RCTs: Randomized controlled trials
SEs: Standard errors
T2DM: Type 2 diabetes mellitus
WMD: Weighted mean difference
