# Supplementary material for: Lack of efficacy of pomegranate supplementation for glucose management, insulin levels and sensitivity: evidence from a systematic review and meta-analysis
Source: Nutr J. 2017 Oct 6;16:67. doi: 10.1186/s12937-017-0290-1 (PMC5629805; doi:10.1186/s12937-017-0290-1)
Supplement: Additional file 1: — GRADE Evidence Profile. (DOC 38 kb) [file 12937_2017_290_MOESM1_ESM.doc]

| **Additional file 1 GRADE Evidence Profile** | | | | | |
| --- | --- | --- | --- | --- | --- |
| **Outcomes** | **No of Participants (studies)** Follow up | **Quality of the evidence** (GRADE) | **Relative effect (95% CI)** | **Anticipated absolute effects** | |
|  |  |  |  |  |  |
|  |  |  |  | **Risk with Control** | **Risk difference with Pomegranate supplementation** (95% CI) |
| **Fasting blood glucose** | 590 (15 studies) 1 to 12 weeks | ⊕⊕⊕⊕ **HIGH** |  | The mean fasting blood glucose ranged across control groups from  **-2.79 to 1.58 mg/dL** | The mean fasting blood glucose in the intervention groups was **0.6 lower** (2.79 lower to 1.58 higher) |
| **Fasting blood insulin** | 388 (8 studies) 1 to 12 weeks | ⊕⊕⊕⊝ **MODERATE**^1^ due to inconsistency |  | The mean fasting blood insulin ranged across control groups from  **-1.16 to 1.75 μIU/mL** | The mean fasting blood insulin in the intervention groups was **0.29 higher** (0 to 1.75 higher) |
| **Glycated hemoglobin** | 163 (3 studies) 8 to 12 weeks | ⊕⊕⊕⊕ **HIGH** |  | The mean glycated hemoglobin ranged across control groups from  **-0.39 to 0.18 %** | The mean glycated hemoglobin in the intervention groups was **0.11 lower** (0 to 0.18 higher) |
| **Homeostatic model assessment of insulin resistance** | 368 (7 studies) 1 to 8 weeks | ⊕⊕⊕⊝ **MODERATE**^1^ due to inconsistency |  | The mean homeostatic model assessment of insulin resistance ranged across control groups from  **-0.53 to 0.46** | The mean homeostatic model assessment of insulin resistance in the intervention groups was **0.04 lower** (0 to 0.46 higher) |
| *The basis for the **assumed risk** (e.g. the median control group risk across studies) is provided in footnotes. The **corresponding risk** (and its 95% confidence interval) is based on the assumed risk in the comparison group and the **relative effect** of the intervention (and its 95% CI). **CI:** Confidence interval; | | | | | |
| GRADE Working Group grades of evidence **High quality:** Further research is very unlikely to change our confidence in the estimate of effect.  **Moderate quality:** Further research is likely to have an important impact on our confidence in the estimate of effect and may change the estimate. **Low quality:** Further research is very likely to have an important impact on our confidence in the estimate of effect and is likely to change the estimate. **Very low quality:** We are very uncertain about the estimate. | | | | | |
